# Utilisation of Electronic Health Records for Public Health in Asia: A Review of Success Factors and Potential Challenges

**DOI:** 10.1155/2019/7341841

**Published:** 2019-07-08

**Authors:** Lesley Dornan, Kanokporn Pinyopornpanish, Wichuda Jiraporncharoen, Ahmar Hashmi, Nisachol Dejkriengkraikul, Chaisiri Angkurawaranon

**Affiliations:** Department of Family Medicine, Faculty of Medicine, Chiang Mai University, 110 Intawaroros Road, Muang, Chiang Mai, 50200, Thailand

## Abstract

**Introduction:**

Electronic health records offer a valuable resource to improve health surveillance and evaluation as well as informing clinical decision making. They have been introduced in many different settings, including low- and middle-income countries, yet little is known of the progress and effectiveness of similar information systems within Asia. This study examines the implementation of EHR systems for use at a population health level in Asia and to identify their current role within public health, key success factors, and potential barriers in implementation.

**Material and Methods:**

A systematic search process was implemented. Five databases were searched with MeSH key terms and Boolean phrases. Articles selected for this review were based on hospital provider electronic records with a component of implementation, utilisation, or evaluation for health systems or at least beyond direct patient care. A proposed analytic framework considered three interactive components: the content, the process, and the context.

**Results:**

Thirty-two articles were included in the review. Evidence suggests that benefits are significant but identifying and addressing potential challenges are critical for success. A comprehensive preparation process is necessary to implement an effective and flexible system.

**Discussion:**

Electronic health records implemented for public health can allow the identification of disease patterns, seasonality, and global trends as well as risks to vulnerable populations. Addressing implementation challenges will facilitate the development and efficacy of public health initiatives in Asia to identify current health needs and mitigate future risks.

## 1. Introduction

The implementation of electronic health records (EHR) in medical practice has seen a significant increase in recent years. EHR systems present a valuable opportunity to improve health surveillance and evaluate service provision potentially leading to improvements in the management and the promotion of public health [1]. Findings suggest that most clinicians use the information available to examine the overall condition of the patient and inform clinical decision making and for shared communication across patient care teams [2]. By June 2013, three-quarters of office-based physicians in the United States had incorporated EHR into their practices [3]. The purchase and implementation of EHR systems are a significant investment of resources but the effectiveness of the approach also depends on the physicians' willingness to adopt the new technology into everyday practices [4].

Primary clinical care and population health have complementary goals of improving the health of patients and communities but seldom create effective partnerships to increase the wellness of both the patient and populations [5–7]. Changing healthcare goals require flexible systems. In the current financial climate, it may be argued that population health requires the proactive application of strategies and interventions to defined groups of individuals to improve the health of those individuals at the lowest cost [8]. Researchers have been using EHR systems to gather rich data in areas such as heart disease, smoking, and the delivery of preventative services [9, 10]. EHR have allowed for the tracking and consolidation of vaccination programmes, enabling improved design and sustainability of effective immunisation strategies [11]. For most healthcare providers, EHR provide easy access to patient information, and although the value of EHR in clinical settings is not to be underestimated, the technological requirements for health information are ever-changing [12]. For example, in the United States, the introduction of the Patient Protection and Affordable Care Act (ACA) was predicted to radically change the functions of health departments, requiring new developments in health technology in an effort to track those changes and potentially creating competitive pressures [13]. Data sharing—in keeping pace with policy changes—brings a new level of complexity. A recent international comparative study of the use of electronic medical records (EMR) for research found that the procedures for information governance, levels of adoption, and required time and ease of obtaining consent varied significantly across the countries [14]. Existing systems for collecting and analysing data frequently lack coordination and effective interconnectedness within the departmental and hospital systems, creating challenges in the analysis and interpretation of patient outcomes, particularly as it pertains to a specific population or community [15–17]. The ability to provide effective and preventative care management will require a more sophisticated and expansive level of data collection on selected populations that currently outstrips the capacity of most healthcare organizations [18].

However, the gap between medical demands and supply also varies significantly between high- and low- and middle-income countries (HIC; LMIC) [17]. The challenge facing public health practitioners is that as EHR and EHR systems progress, the gap between high- and low- and middle-income countries widens, increasing the risk that the most vulnerable populations are left further behind in the provision of effective healthcare and public health strategies. While developed countries such as the United States and United Kingdom have led the way in the implementation of EHR, less is known of the progress and effectiveness of similar information systems within Asia. Understanding the progress that has been made and the processes by which EHR is adapted to different settings in Asia allows practitioners an opportunity to learn valuable lessons and implement effective systems to promote and improve individual and community health.

Therefore, this review examines the implementation of EHR systems for use at a population health level in Asia and to identify their current role within public health, key success factors, and potential barriers in implementation.

## 2. Material and Methods

### 2.1. Analytical Framework

**Figure 1:**
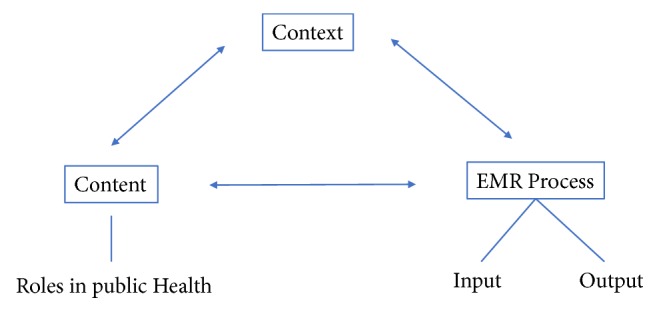
Analytical framework exploring the role of electronic health records in public health in Asia.

The analytical framework for this review was adapted from studies examining the impact of EHR in medical office settings and a conceptual framework for data visualisation using EHR [19, 20]. In order to examine the content for utilisation in public health, one needs to consider it along with two other interactive components: the context and process [19]. The context can be further classified as internal and external. For the purposes of this review, the internal context refers to the structure, culture, and resources of the organization utilising the EHR. The external context refers to the larger socioeconomic and political environment in which the organisation operates. For this review, “process” is classified into the input process and output process [20]. The input process considers all factors related to data entry, which may consider cultural factors or available resources. Output process considers all factors related to data visualisation and its output (Figure 1).

### 2.2. Search Strategy

A systematic search process was completed to identify relevant articles related to utilisation of EHR for public health in Asia. Specifically, articles to be included must fulfil two key components:

1. The article must
  - be based on hospital/service provider electronic records
  - AND
  - have a component of implementation, utilisation, OR evaluation
2. The article must be related to public health by fulfilling one of the following criteria:
  - Going beyond direct clinical or patient care
  - OR
  - Being health systems related

Electronic records or systems not related to public health as well as summary or opinion papers, abstracts, news articles, and reviews were excluded. Medical Subject Headings (MeSH) were identified and used as search terms including “information systems,” “database management systems,” “medical record systems,” “hospital information systems,” “information technology,” and “software, software design, and software validation.” MeSH keywords fitting outcomes of interest included “decision making,” “health planning,” “health policy,” “public health,” “systems integration,” and “organisational culture.” Finally, these terms focused on Asia with articles published from January 2008 to May 2019. A total of five databases were searched: CINAHL, EMBASE, Medline, Web of Science, and PubMed.

For each study, we extracted its current content and utilisation in public health and key information including its aim, methods, findings, and limitations as identified by the original authors of the studies (Appendix Table 1). Key success factors and barriers to implementation as identified by the original authors were extracted. Relevant information from each study was classified into two key components outlined above: context and process (Appendix Table 2).

### 2.3. Search Outcomes

**Figure 2:**
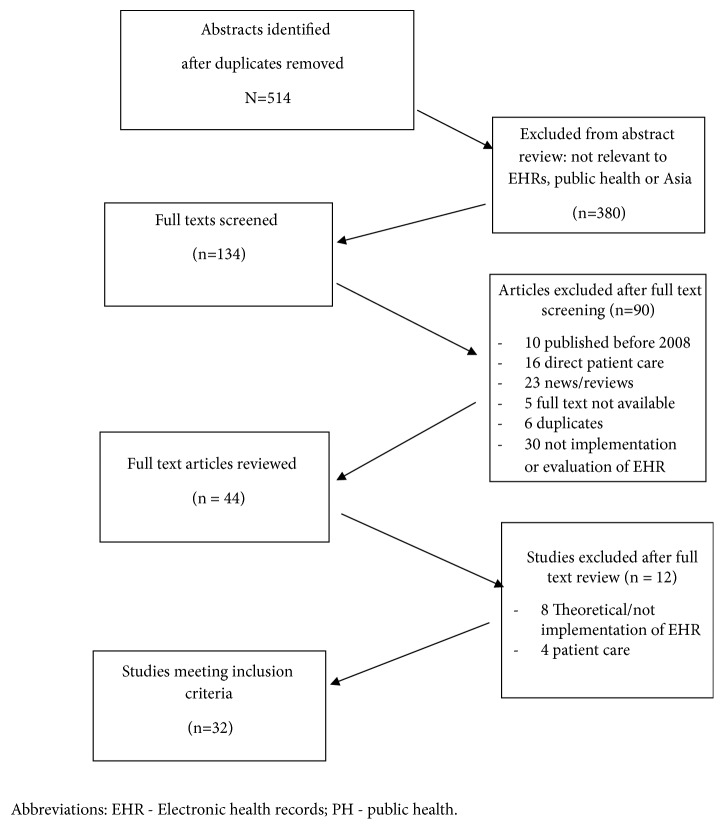
Flow chart of studies examining the role of electronic health records in public health in Asia.

Following searches in all the named databases, a total of 465 articles were identified. To ensure identification of all relevant articles, the initial search focused on all articles including EHR and/or Asia and/or public health related activities. Specific details have been summarized in Figure 2. Four researchers (LD, WJ, AH, and CA) performed the abstract reviews and assessed the full texts. Six researchers (LD, WJ, KP, AH, ND, and CA) were responsible for data extraction of the included reviews. In addition to following the analytical framework outlined above, each paper was examined for common themes associated with challenges and good practice. A thematic analysis as guided by the framework was applied and performed by three investigators (LD, AH, and CA), with extracted data compiled and analysed using NVivo 12 (QSR International, Doncaster, Victoria, Australia).

## 3. Results

A total of 32 studies (Figure 2) were included from 15 countries and/or regions, including one study from multiple cities across Asia, one study reporting from Africa and Asia, and Singapore (n=6); China (n=4); Iran (n=1); Malaysia (n=3); Thailand (n=3); Indonesia (n=2); Myanmar (n=2); South Korea (n=2); Taiwan (n=3); India (n=1); Japan (n=1); the Philippines (n=1); and Vietnam (n=1). The studies included in this review reflected both the complexity of this field of research and the breadth of practice within the public health discipline. In addition, these studies come from highly variable contexts with respect to the “maturity” of the electronic systems and socioeconomic differences as they relate to technological and health systems infrastructure. Studies reviewed here also occurred in different contexts and at different levels of a health system, including research across international contexts (n=3); at national levels (n=7); at provincial or state levels (n=4); across organizations (i.e., nongovernmental organizations; n=2); at the district, community, or village levels (n=4); and in tertiary care facilities (n=13).

Public health research being carried out within Asia included preparedness for pandemics, communicable and infectious diseases such as leprosy, sexual health, maternal health, and cancer (Appendix Table 2). It also incorporated evaluations of systems already in place in urban and/or rural regions ranging from primary to tertiary care as well as across different health care providers, such as nongovernmental organizations.

### 3.1. Role and Benefits of Electronic Health Records to Public Health

**Figure 3:**
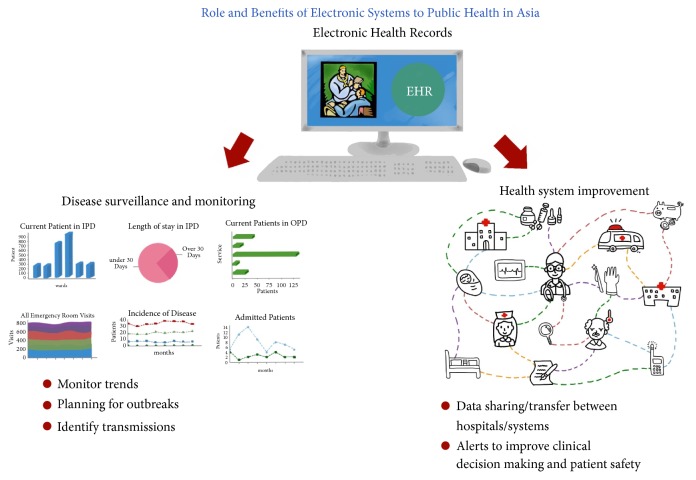
Role and benefits of electronic health records to public health in Asia.

It was clear from several of the studies that—while recognizing difficulties in integration and development of EHR within Asia—there were also significant benefits. The benefits of leveraging electronic systems focused primarily on disease-, patient-, or situation-specific interventions as well as improvement of “systems-level” functioning, or both (Figure 3). A key element of public health in Asia is the utilisation of EHR for disease surveillance and monitoring systems. EHR have the ability to help identify and predict seasonal outbreaks and high risk areas and prevent infections or diseases as well as assisting in the coordination of demographic information and community profiles, which are invaluable in the current public health climate. However, concerns about confidentiality were noted [21–26]. Another key utilisation of EHR is their implementation to improve health care systems. The identification of risk factors through electronic health systems allows health professionals to recognize and track them over time, helping both in clinical decision making, planning for outbreaks, and identifying transmission of diseases [27, 28]. For example, a study of cancer patients allowed the tracking and analysis of diagnostic patterns, the number of investigations completed by physicians, and transfer of information as well as factors for the diagnoses [29].

### 3.2. Success Factors and Potential Barriers

#### 3.2.1. Context

Of the studies reviewed, 26 of 32 noted external contextual factors, from all countries represented across all studies. Of all studies, 17 commented on internal contextual factors within the system the study was conducted; and 15 studies had commentary on both external and internal contexts. External contextual challenges often related to the wider infrastructure, such as variability in contexts relating to centralisation of information and human resource and information and communication technology (ICT) constraints [21, 25, 26, 30–34]. For example, Kimura et al. observed healthcare system issues arising during the implementation of EHR for intractable diseases in Japan [30], where a complex, decentralized administrative system and language barriers related to the Japanese script required country-specific tools and expertise to overcome data entry challenges. A study from Taiwan explored ways to overcome the challenge of data exchange between hospitals [34]. A lack of funds for healthcare technology as well as a lack of public health government initiatives and a fragmented healthcare system also created challenges in health care provision [21, 33]. Access to mobile networks and web-based technologies demonstrated the variability in “maturity” of the different contexts, where important constraints particular to LMIC contexts were observed in three studies conducted in India, Myanmar, and China. In these contexts, inconsistent power supplies led to difficulties in EHR system implementation and intermittent internet availability constrained the development of web-based services [21–23]. However, a number of studies were able to leverage mobile networks and web-based platforms to wider benefit [24, 35–38]. Studies reported different levels of maturity vis-à-vis EMR systems reach within a given context [27, 29, 39–46]. However, studies conducted during earlier stages of EMR introduction documented progression from paper to electronic documentation as particularly time-consuming and requiring significant human resource allocation [21, 22, 47]. In rural China, for example, data entry was required to transfer data from paper-based systems to web-based forms by on-site staff or through instructions from mobile phone conversations, landlines, or fax, which carried a higher risk of human error requiring data entry supervision [22].

Internal contextual factors were often couched within the larger, external context, but specifically related to an organisation's local access to ICT support [27, 47]; human resource needs in transitioning from paper to electronic records [23, 28, 31, 38]; local access to existing systems at higher levels, i.e., national/provincial/state infrastructure, web-based platforms [35, 39, 43, 45]; and locally existing (or lack of) EMR systems [29, 41, 45, 46, 48–50].

#### 3.2.2. EHR Input Process

All studies (n=32) reported elements of the EHR input process. Several of the studies highlighted the importance of internal organisational cultures and the impact this had upon the EHR input process. Key areas of intervention or identifying potential EMR inputs was related to conceptual approaches [37, 46] or cultural considerations [27, 30, 51]. Infrastructural considerations related to hardware or workforce training were noted in studies by Herbst et al. and Sutiono et al. [32, 37]. Specifically with regard to the workforce, many studies assessed end-user (or potential users) evaluation of previously implemented or planned ICT interventions [23, 31, 38, 42, 43, 45, 46, 48, 50]. Multiple studies highlighted interventions based around software, web-based platforms, or mobile technologies [21, 24, 25, 36, 37, 46]. Specific ICT interventions incorporated elements of automation [28, 30, 44, 49, 52]; data standardization or quality control [17, 22, 24, 31–33, 35, 47]; data visualisations or data mapping [21, 35, 37, 38, 44, 45, 49]; and data analysis tools [22, 24, 29, 39–41, 46]. Finally, some studies also specifically mentioned measures protecting patient information [17, 33, 36, 38].

#### 3.2.3. EMR Output Process

Most studies (n=31) reported EMR outputs for the various ICT interventions covered. Disease-specific recommendations were made in three studies from China [27], Indonesia [25], and Taiwan [52]. Workforce and human resource considerations were reported in several studies [23, 42–45, 50], particularly with regard to transition from older electronic and computerized systems to more technical interfaces and tracking systems. Several studies highlighted important recommendations related to outputs and visualisations such as standardization of unique patient identifiers; modular, flexible information systems structures; bilingual and user-friendly interfaces; and ease of uploading and sharing important clinical information based on the authors' findings [26, 31, 44, 47, 48]. Clinical and health dashboards constituted a common intervention [22, 33, 35, 37, 40, 46, 52], with additional studies also incorporating automated alert systems [28, 29, 45], and a number of studies focused on data analysis and public health reporting [21, 24, 25, 30, 32, 36, 38, 40, 41, 52]. Finally, studies in Taiwan [27]; Africa and Asia [32]; South Korea [53]; and China [47] documented the creation of online tools and data repositories as a result of their respective interventions.

## 4. Discussion

This review summarises efforts to implement EHR systems for use in different capacities in Asia. We highlight 32 studies conducted in 15 countries with two studies comparing sites across countries in Asia. This review compiles information on EHR systems across a diversity of country and healthcare contexts including LMIC settings, varying organisational structures and different levels within health systems. It represents varied technological infrastructure and EHR system “maturity” and their resultant human resource needs.

This review highlights challenges that exist in utilising EHR systems to improve public health in Asia. Highly variable infrastructural constraints related to supporting EHR systems (e.g., reliable electricity and mobile technologies) add a layer of complexity in terms of system requirements and the level of EHR sophistication that can be supported. Therefore, within a given context, risks may be inherent in introduction of EHR for use in public health. Barriers of note relate to the organisational culture and highlight the need for well-trained technological support in healthcare settings in Asia. Hospitals frequently find that delays in EHR implementation can occur due to the nonadoption of the system by physicians and health professionals [54]. A study in Iran identified that organisational barriers in the implementation of EHR included a lack of efficient planning, a lack of skilled manpower, and limitations in information technology training for healthcare professionals [55]. Given these concerns, ways forward would include* a priori* evaluations of organisational cultures and settings where EHR systems are introduced that assess the required technical support; explore staff awareness, skill levels, and willingness to utilise new technologies; and evaluate current data collection methods in an effort to stymie early barriers to implementation. Addressing staff concerns of using new ICT interventions prior to implementation can prevent reluctance to adopting new practices, as well as allaying concerns regarding the management of and workloads associated with the new system. Such explorations may help with implementation within a given health system or across an organisation, allowing a more tailored approach to EHR interventions that are contextualised based on specific externalities that may pose barriers but cannot be effected at the level of implementation.

In addition to technological and practical risks, studies also highlighted ethical concerns in introducing EHR interventions. As EHR become more commonplace in LMIC settings, on-going debates in HIC regarding patient confidentiality, privacy, informed consent, and data security remain salient in resource-poor contexts [56]. This reflects that, globally, EHR systems are developing at a rapid rate and in a manner that may outstrip the ability of LMIC contexts to manage such concerns arising from EHR implementation. Many smaller healthcare providers and individual hospitals are still looking to implement effective EHR systems or convert from disparate applications provided by multiple suppliers to an effective, unified system [57]. Merging such systems in LMIC requires careful consideration of patient-provider interactions that include cultural appropriateness, ethnic health disparities, low levels of patient literacy, linguistic challenges, and necessary institutional oversight of the patient-provider relationship [56]. Ways forward would include more systematic, comprehensive preparations prior to implementing effective and flexible EHR systems to meet public health needs. As with technical and practical barriers observed by introducing EHR interventions, considerations of ethical issues are also integral in the successful implementation of effective EHR programmes. Providing the context for EHR implementation and formal instruction of ethical risks should provide health care professionals and support staff with means of mitigating patient risks.

This study reflects findings from other reviews of the use of EMR/EHR in LMIC settings. Williams and Boren point out that most developing countries have constraints often external to the health systems within which EMR/EHR are implemented, such as infrastructure and energy constraints [58]. With a focus on quality of health data and health information management, a review of community and district levels in LMIC outlined poor quality data, poor management of hospital information systems, and low utilisation of health information as the predominant barriers to implementation [59]. Another insightful review recognised implementing EMR interventions in LMIC as an “evolving,” long-term process, with no comprehensive blueprint for a given health system, when considering the complex social systems encompassing such interventions [60]. Applying a stakeholder perspective, Akhlaq et al. concluded that higher societal level factors, such as political will and financial commitments, were integral to wide-scale hospital information exchange improvements [61]. In addition to substantiating many of these findings from other LMIC settings, this review adds an Asian focus that also allows a comparison across varying health systems across a broad region, focuses specifically on EMR for public health interventions, and highlights factors both within organisations and external to an organisation in implementing EMR interventions.

This study has some limitations. As only articles published in English were included, it is possible that some studies from the region may have been omitted if published in local languages. However, thematic analysis of data obtained from the 32 studies determined recurring themes that were corroborated by multiple researchers, suggesting robust analysis. The overall findings suggest that the benefits of EHR in public health should far outweigh the challenges faced in the region. An international comparative study including China, Indonesia, Taiwan, and India suggested that the adoption of EHR had considerable potential to improve the safety, quality, and efficiency of healthcare as well as being a valuable resource for research [14]. For efficient implementation of systems and utilisation of data for public health and research, an effective collaboration of academia, regulated industries, policy makers, patients, and health professionals is critical [62]. The lack of interoperability between systems requires an effective, unified information system and can prove to be a major roadblock to those attempting to move healthcare forward to an integrated system of care [63]. The process of moving from a paper-based to an electronic database system and subsequently to a platform or web-based scheme can be arduous. The knowledge, expertise, and software required for these systems can be a challenge but with the increased reach of the internet, resources can become available. The selection of systems best suited to meet an organization's needs and define the implementation plays a critical role in the success of a given EHR project [64]. However, there is a potential need for long-term and systematic funding to develop nationally or regionally integrated systems.

## 5. Conclusion

The progress and capacity of EHR systems is far-reaching and effective. Understanding broader and local contexts, access to available resources, addressing organisational challenges, and implementing well thought-out approaches in the development of EHR projects should go a long way to address potential barriers to EHR implementation. The values of EHR are significant and go beyond individual clinical decision-making in its ability to identify disease patterns, seasonal and global trends, and the potential risks to vulnerable populations as well as to strengthen coordination of care between different sectors. Understanding the potential capabilities and preparing for potential challenges of EHR as highlighted in this study will help facilitate the development and implementation of public health initiatives in Asia to address current needs and identify future risks.

## Data Availability

All data generated or analysed during this study are included in this publish article and its supplementary information files.

## Abbreviations

EHR: Electronic health record
ICT: Information and communication technology
HIC: High-income country
LMIC: Low- and middle-income country.

## Supplementary Materials

Supplementary Materials Appendix Table 1. Summary of articles included in the review. Appendix Table 2. Summary of thematic analysis of articles included in the review.
