# Supplementary material for: Utilisation of Electronic Health Records for Public Health in Asia: A Review of Success Factors and Potential Challenges
Source: Biomed Res Int. 2019 Jul 8;2019:7341841. doi: 10.1155/2019/7341841 (PMC6644215; doi:10.1155/2019/7341841)
Supplement: Supplementary Materials — Appendix Table 1. Summary of articles included in the review. Appendix Table 2. Summary of thematic analysis of articles included in the review. [file 7341841.f1.docx]

**Appendix Table 1**. Summary of articles included in the review

| **Authors & Title** | **Aim** | **Methods** | **Findings** | **Limitations** |
| --- | --- | --- | --- | --- |
| **Alipour et al (2017)**  Success of failure of hospital information system of public hospitals affiliated with Zahedan University of Medical Sciences: a cross sectional study in Southeast of Iran **(Iran)** | Evaluate success or failure of hospital information systems in public hospitals affiliated with Zahedan University of Medical Sciences. | Cross sectional study conducted in 2016. Three groups surveyed: information technology and hospital information system authorities, senior health managers and hospital information system (HIS) users | HIS relatively successful in terms of functional, ethical and cultural factors but were considered as a relative failure in terms of behavioural, organisational and educational factors from users’ perspective. Only legal factors showed success from HIS and IT authorities’ perspective | Lack of manager’s participation in this research |
| **Baldwin et al (2008)**  Managing information: using systematic data collection to estimate process and impact indicators related to harm reduction services in Myanmar  **(Myanmar)** | Discusses the process involved in developing a management information system in Myanmar and potential ethical implications of the proposed data collection system for harm reduction interventions. | A case study summarizing the development of a coordinated system among several partner agencies capable of measuring collective coverage of harm reduction services.  Series of workshops were conducted with partner agencies: identified best practices, mapping existing local harm reduction policies and procedures; refined MIS functional requirements, data collection instruments and user interface designs; developed prototypes and a beta version to be field tested. | Outlines key features of MIS: unique identification codes; configuration conducive to optimal flexibility at site level/agency; bilingual user interface with simple data analysis and reporting functions. |  |
| **Chang et al (2015)**  A context aware approach for progression tracking of medical concepts in electronic medical records  **(Taiwan)** | To introduce a context-aware approach to assign the time attributes of recognised risk factors by reconstructing contexts that contain more reliable temporal expressions. | “Context-aware” implies an algorithm that effectively dates the occurrence of medical concepts related to disease. After identifying clinical text within electronic documents, medical concept recognisers processed disease mentions, along with risk factors and medications. A time-attribute assigner component used the proposed context-aware algorithm, to analyse textual information according to two different context range parameters.  The new algorithms were compared against a baseline system (cTAKES) on a broad dataset. | The context-aware approach achieved better precision in assigning time attributes among all concept types compared to the without context-aware approach with a better overall F-score (0.882 vs 0.827). The system proposed a context-aware approach to track progression of the medical concepts. | Certain types of medical concepts may not have explicit temporal expressions and so the algorithm assigns the most common time attribute. Baseline system did not benefit from annotated corpus and may result in simplified feature sets. Need for classifiers/ID for patients, risk factors and diseases and recognised concepts. |
| **Eunmi et al (2016)**  The automated alert system for the hospital infection control and the safety of medical staff based on EMR data  **(South Korea)** | Report on the planning, development and implementation of an automated alert system for hospital infection control. Specifically considered how to increase the usability of the system and how to enhance data quality. | Formation of a task force for new infection alert program using existing EMR. Defined data rules, designed user interface and considered usability of the system. Included infectious diseases, rules for updating system corresponding to lab results and rules for data migration. | 17 infectious diseases were defined within the system; included various types of transmission; automatically registered patients according to lab test results; and released patients from alert system based on subsequent lab testing/discharge, with manual override. Designed user interface for infection control and updating system as well as data migration for past records. System appeared to be accurate and convenient. | Data updates are not in real time, only once every night (to avoid system overload). |
| **Herbst et al (2015)**  The INDEPTH data repository: an international resource for longitudinal population and health data from health and demographic surveillance systems  **(Africa/Asia)** | Summarizes the International Networks for the Demographic Evaluation of Populations and Their Health (INDEPTH), a global network of research centers that conduct longitudinal health and demographic evaluation of populations in low- and middle-income countries. | Summarize different elements of the system database including: data resources needed (including support); data production process (conceptual development; data management workshops; quality assurance); and data resource use (i.e., access). | Core micro datasets on the repository with data from 25 centers representing 2 million individuals and 24 million person years of observation, including 110 dataset downloads from the Asia region (Vietnam; Thailand; Indonesia).  Comprises the largest dataset on cause specific mortality in LMICs.  Can be used for assessing population impact of infectious disease and for quantifying MDG.  Data production support elements: two, staffed support nodes in Africa and India; “Centre-in-a-Box” (CiB) which includes an all-in-one computer hardware and software environment distributed to participating centers. Data production process: conceptual development; data management workshops; quality assurance. | Limited scope due to the small number of sites providing datasets.  Not technically representative of given populations. |
| **Jinpon et al (2017)**  Integrated information visualization to support decision-making in order to strengthen communities: design and usability evaluation  **(Thailand)** | Integrate visualisation and explored applicability, in the form of Community Well-Being Assessment System (CWBAS) as a web-based tool for the querying, analysing, and visualising of patterns of community well-being in Thailand. | Cross-sectional descriptive design.  Focused on 10 categories and 50 indicators for community well-being assessment.  Calculated community well-being scores comparing existing questionnaire data in 9 sub-districts across the 4 regions in Thailand.  Computer program developed to standardise performance indicators and represent statistics graphically.  Effectiveness and user perception of CWBAS evaluated through the use of questionnaires for 243 users in three sub-districts at a variety of positions across sub-administrative offices and health centers as well as villagers. | Community well-being scores averaged and fell within the range of 0.00-3.00. Community well-being scores highest in Southern region (2.05), followed by the Northern region (1.98), Central (1.93) and lowest in the Northeastern region (1.74).  Overall average score for user satisfaction was 4.12 on a 5-point Likert scale. GIS analytic tool rated as the most useful feature in the CWBAS. Satisfaction with system performance and security received an average score of 4.06 and satisfaction with system process a score of 4.21. | Potential selection bias in evaluation of CWBAS as participants selected by chief executives and heads of villages. Potential bias for inflating positive reviews of health and administrative staff level participants, who fill positions best equipped to utilize CWBAS. |
| **Kimura et al (2011)**  Developing an electronic health record for intractable diseases in Japan  **(Japan)** | Feasibility study of electronic health record (EHR) to examine the possibility of an EHR programme for intractable disease specific to Japan. | Defined intractable disease as resulting from unidentifiable causes, are very difficult to treat, or for which no treatment procedure has been established. Methods examined the EHR standard adaptation by identifying intractable disease through defined selection criteria. Developed clinical research templates based on 6 diseases. Assessed compatibility with current administrative procedures and the clinical research form entry system that allows health information to be stored and sensitive patient data to be anonymised. | 49 archetypes were designed to define the templates. Embedding codes in traditional research forms reduced large scale administrative changes and improved operating effectiveness and quality of data. Study suggests that approach could build a system that keeps personal information private while allowing collection of data. | Japan has a decentralised system, complex administrative procedures which causes difficulties. Challenges included linguistic issues and a lack of global unique identifiers such that patient information may overlap. Needs country specific additional tools. Previous system not digitised and data entered manually, resulting in low quality data. |
| **Lee et al (2015)**  Validation for accuracy of cancer diagnosis in electronic medical records using a text mining method  **(South Korea)** | To validate the accuracy of cancer diagnosis data in existing EMR using a text mining method. | Compared diagnosis of cancer patients with pathology reports. Categorised 14 types of cancers based on physician diagnosis codes. Compared diagnosis to a text search for pathologic reports. Correct diagnoses were defined as one or more pathology reports matching the clinical diagnosis. | Two-thirds of 236,012 cancer patients with at least one pathological examination were filtered. Concurrence between diagnosis and pathology report was highest in thyroid cancer patients (86.3%) and lowest in liver cancer patients (49.9%). Recommends a systematic, clinical decision support system to prevent errors in data input. | In liver cancer, most diagnoses are made by history of underlying liver disease and the result of imaging. |
| **Li et al (2013)**  e-Health preparedness assessment in the context of an influenza pandemic: a qualitative study in China  **(China)** | To assess the preparedness status of a hospital in Beijing for implementation of an e-health system in the context of a pandemic response. | Qualitative approach in two phases. 1) Group interviews conducted with key stakeholders to examine the effectiveness of the surveillance system with information and communication technology;  2) individual interviews to gather data in relation to e-health preparedness within the hospital system. | Measures included motivational forces for change, healthcare provider’s exposure to e-Health, technological preparedness, organizational non-technical ability to support a clinical innovation and socio-cultural issues with pandemic responses. Major issues identified included poor sharing of patient health records, errors, unavailability of software tools, concerns of reliability of the system and cost. | Limitations may include over-reporting or recall bias. |
| **Liabsuetrakul et al (2016)**  Development of web-based epidemiological surveillance system with health system response for improving maternal and newborn health: field-testing in Thailand  **(Thailand)** | Developed a web-based epidemiological surveillance system for maternal and newborn health with integration of action-oriented responses and automatic data analysis with results presentations. Also assessed system among health workers in various hospitals in southern Thailand. | Developed and tested system across eight hospitals in Songkhla province, southern Thailand.  Trained one or two labor and delivery nurses and one obstetrician from participating hospitals.  Two smartphones deployed: one available in delivery rooms for data entry into trial system. A second smartphone given to trained doctors responsible for action-oriented responses by reviewing indicator events. Administrator continuously monitored data entry at a data center.  System evaluated after field-testing ended through anonymous evaluation forms to all doctors and nurses participating in the system.  Outcome measures included: completeness of data entry; acceptance of system through perceived ease of use; and intention to use the trial system. | 2459 deliveries recorded, 920 women with at least 1 of 10 complications monitored by surveillance system. 2 obstetricians, 3 general practitioners, and 40 nurses involved in the data entry process assessed by the system.  Half of all participants reported the system as easy or very easy to use. 2/3 reported the system beneficial to strongly beneficial. Rate of intention to use the system ~40%. Reasons for reluctance included: current workload for data entry within 2 h of delivery and monitoring complications and 24 h after delivery; perceived ease of use related to intention to use. | Does not assess impact of system on health outcomes or survival.  Potential for under-report of complications from childbirth.  Data could be disaggregated by only a limited number of variables.  System limited in areas where quick and uninterrupted internet access cannot be ensured.  Social influences and/or subjective norms were not tested. |
| **Lo et al (2014)**  Improving the work efficiency of healthcare-associated infection surveillance systems using electronic medical records  **(Taiwan)** | Development of an integrated hospital-associated urinary tract infection surveillance information system based on electronic records to improve the work efficiency of Infection Control Professionals (ICP) and create a common system for implementation. | An integrated hospital culture based associated urinary tract infection surveillance system was developed based on existing EMR. A model was designed to collect data from the different systems and provide a visualisation dashboard to facilitate planning and surveillance. Comparison was made with an alternative system. A three-state system was developed to identify cases and notify ICP as required. Changes are made by the ICP. | The model showed a reduction in ICP time costs and better access through the implementation of a web-based service which could be accessed by laptops or mobile technology. In addition, the newer system allowed for increased sensitivity in determining cases with feedback to ICP for further investigation. | The EMR systems did not support all surveillance data. Therefore, each patient’s state and the ICP usage of the system varied causing time differences between cases in manual chart review. |
| **Low et al (2017a)**  Evaluation of a practical expert defined approach to patient population segmentation: a case study in Singapore.  **(Singapore)** | Assesses the validity and feasibility of the proposed segmentation framework to categorize the patient population into distinct, non-overlapping patient segments. Describes patient profile and health utilisation in each segment. | Retrospective, cross-sectional study segmenting 2012 SingHealth RHS patient populations. Analysed variables from socio-demographic, chronic diseases and prior healthcare utilization. Categorised chronic disease by those with or without frequent hospital admissions (≥3 hospital admissions). | Population database of 825,874 adult patients with mean age of 55.6 years. Healthcare utilisation highest among patients with complex chronic disease with frequent hospital admissions, with “end of life” segment accounting for highest specialist clinic visits and highest mortality rate (33%). | Variables restricted to those routinely collected in EHR and administrative databases, which did not allow for refining segmentation by patient functional status, caregiver availability or degree of social support needed.  Did not include private sector patients.  Databases unable to account for cross-utilisation of healthcare services outside SingHealth RHS or out of hospital deaths. |
| **Low et al (2017b)**  FAM-FACE-SG: a score for risk stratification of frequent hospital admitters  **(Singapore)** | To derive and evaluate a risk stratification tool to predict frequent hospital admitters. | Retrospective cohort study from EHR of tertiary hospital in Singapore.  Primary outcome: three or more inpatient readmissions within 12 months of index discharge.  Univariable and multivariable logistic regression models built frequent hospital admission risk scores (FAM-FACE-SG) incorporating demographics, socioeconomic status, prior healthcare utilization, markers of acute illness burden, and markers of chronic illness burden.  Validated risk score by comparison to LACE index with receiver operating characteristic analysis. | Included 25,244 patients with 70% randomly selected patients for risk score derivation and 30% of patients used for score validation.  Found 17.1% of patients with three or more inpatient readmissions within 12 months of index discharge.  FAM-FACE-SG score consisted of 9 components with good discriminative ability with AUC of 0.839. LACE, by comparison, achieved AUC of 0.761. | Datasets restricted to those routinely collected in administrative and clinical databases.  Excluded predictors such as caregiver availability and function.  Bias in modeling may be due to not excluding patients who may have died after index hospital discharge. |
| **Low et al (2017c)**  Performance of the LACE index to identify elderly patients at high risk for hospital readmission in Singapore.  **(Singapore)** | Assess performance of LACE index in Singaporean cohort and investigated the use of additional risk factors in improving readmission prediction performance. | Retrospective analysis of EHR data collected from Singapore General Hospital in 2014, through the SingHealth registry. | 17,006 patients analyzed with 12.1% having a 30-day readmission.  LACE with poor discriminative ability, with c-statistic of the LACE index and logistic regression model when augmenting risk factors for LACE at 0.595 and 0.628, respectively. | Prediction model derived from administrative data.  Selection of variables confined to those routinely collected in the administrative database.  Cannot substantiate deaths or 30-day readmissions to hospitals other than SGH.  Generalisability of Singapore-specific data to other contexts. |
| **Mohd Salleh et al (2016)**  The influence of system quality characteristics on health care providers’ performance: empirical evidence from Malaysia  **(Malaysia)** | Assess system quality factors to predict the performance of electronic health in a single public hospital in Malaysia. | Adopted the EHR System Effectiveness Model with four latent constructs to evaluate the performance of the care provider. Four constructs included: adequate infrastructure, system interoperability, perceived security control, and system compatibility.  These constructs were tested via questionnaires given to health care providers in the largest public hospital in northern Malaysia.  Convergent validity and discriminant validity required to evaluate reflective measurement model.  Structural models were constructed and tested to test the 4 constructs and their effects on provider performance. | 46% of the variance in provider performance explained through exogenous, 4 constructs. Every construct’s average variance extracted (AVE) was above 0.5, with the strongest constructs being adequate infrastructure (0.823) and perceived security control (0.759). | Limited integration and interoperability for supporting clinical operations among other Ministry of Health (Malaysia) hospitals, health centers, and clinics.  Limited across disciplines and specialties working within hospitals and with EHR systems. |
| **Murai et al (2011)**  Systemic factors of errors in the case identification process of the national routine health information system: a case study of Modified Field Health Services Information System in the Philippines  **(Philippines)** | Investigates the mechanisms of errors in the case identification process in the existing routine health information system (RHIS) in the Philippines by measuring risk of errors for health program indicators. | Used a structured questionnaire of 12 selected indicators of the Field Health Services Information System (FHSIS) administered to 132 health workers in 14 selected municipalities in the province of Palawan, Philippines. Evaluated health worker understanding of the indicators.  Evaluated difficulty and discriminatory indices. Difficulty index measured proportion of respondents with correct answers to the questionnaire. Discriminatory index measured disparity in the level of understanding of indicators among the health workers. | Discrimination index of 0.216 (p<0.05) when comparing upper group (those scoring the highest) versus the lower group (those scoring the lowest).  Average difficulty index of 12 questionnaire items of 0.266.  Highlights health worker confusion around definitions which may reflect errors in coding. | Generalizability to the rest of the Philippines.  No systematic evaluation of supposed discrimination/difficulty index and their relation to actual coding errors.  Study did not adequately assess years of experience or formal/informal training of health workers.  Study focuses on compliance with current conditions of the systems standards rather than best fit to standard use of data. |
| **Nguyen et al (2013)**  A method to manage and share anti-retroviral (ARV) therapy information of human immunodeficiency virus (HIV) patients in Vietnam  **(Vietnam)** | Describes a system that manages and shares antiretroviral therapy information of 4,438 HIV patients in three healthcare centers in Hanoi, Vietnam. The overall design considerations, architecture and the integration of centralised database and decentralized management for the system are also presented. | Description of system. | Findings from this study can serve as a guide to consider in the implementation model of health care to manage and share information of patients through the process of:   1. Standardization of template and data format; 2. Backup, recovery and security plan; 3. Implementation (user-friendly interfaces). | Although this paper focused on sharing and managing HIV patient’s information, this model can be followed as a general guideline when looking for a methodological approach for the development of similar systems in a different context such as systems to manage information on chronic diseases like cancer, diabetes. |
| **Rachmani et al (2013)**  Health information system model for monitoring treatment and surveillance for leprosy patients in Indonesia (case study in Pekalongan District, Central Java, Indonesia)  **(Indonesia)** | To determine difficulties in a leprosy control system and how a HIS may assist the programme. | Data was collected through in-depth interviews, group discussion and observation of leprosy supervisors and health care workers. Observations were designed to capture workflow, procedures and documentation. | Leprosy data is currently collected by manual records with data compiled in Microsoft Excel. This causes problems with patent management and monitoring. There is a need for computerised databases including data collection, reports and analysis as well as estimates and predicted trends. | Qualitative assessment of existing monitoring system for leprosy. Although a case made for management through improved electronic system, doesn’t include estimates of HR inputs necessary and infrastructural/HR issues at local health centre levels. |
| **Radhakrishna et al (2014)**  Electronic health records and information portability: a pilot study in a rural primary healthcare center in India  **(India)** | To evaluate the feasibility of implementing an EHR system and deploying a data portability solution using a USB-based health card and SMS messaging service. | Study was conducted at a community health training center in Bangalore, India. Participants from the geriatric clinic and child and maternal health clinic were selected. Workflow patterns were studied and interviews with physicians and HCW’s were conducted as well as village leaders. A browser-based EHR, USB memory card and interactive SMS feature were designed. | 233 participants were in the geriatric cohort and 75 in maternal cohort (pregnant and post-natal mothers).  The EHR collected, stored and retrieved patient details and created a community-level database including demographics and medical profiles. Compliance with the health cards took time but allowed regular updating of information. Epidemiological data collected was of considerable value to the health professionals and overall public health picture. Data was accessible for review and analysis. | Frequent power shortages caused problems within the system and caused issues of reliability and availability. Local beliefs also caused some challenges as technology was perceived to negatively affect patients’ faith in the clinician.  Data portability model is unidirectional in only allowing visualisation of data. Data had to be completed at point of entry. No mechanism existed to retrieve immunisation status and anthropometric data within the mothers’ card. Availability of USB cards was an issue thereby limiting data collection. Use of the health cards and SMS system was limited, however, health cards used more frequently after awareness raising activities were conducted. |
| **Rahman et al (2015)**  Emergency medical services key performance  measurement in Asian cities (Tokyo, Osaka, Singapore, Bangkok, Kuala Lumpur, Taipei and Seoul)  **(Asia)** | To compare Emergency medical services (EMS) performance index based on the structure, process and outcome analysis. | Survey created and sent via email to EMS agencies or directors of the Pan-Asian Resuscitation Outcome Study (PAROS) group. Data collected comprised structure, process and outcome information of the EMS system within each EMS agency.  24 variables assessed and compared, but only included variables shared across all EMS agencies in the analysis. | Non-uniformity in the EMS performance measurement across Asian cities. |  |
| **Samy et al (2010)**  Security threats categories in healthcare information systems **(Malaysia)** | Investigate the various types of threats that exist in healthcare information systems in a government hospital. | In-depth, structured interviews of 16 participants (11 from Technology Department; 4 from medical record department and 1 from x-ray department). | Five critical categories of threats   1. Power failure/loss 2. Acts of human error 3. Technological obsolescence (outdated hardware, software, applications) 4. Hardware failure or errors 5. Software failures. | Studied in only one setting using a specific information system. |
| **Sutiono et al. (2010)** Designing an Emergency Medical Information System for the early stages of disasters in developing countries: the human interface advantage, simplicity and efficiency  **(Indonesia)** | Propose how to design the user interface to support emergency medical care in early stages of disasters. | Description of process to determine minimum contents required to define a user interface for critically ill patients. Conducted design analysis and tested against realistic scenarios. Outlines prototype systems architecture. Tested in a disaster simulation area in a remote, difficult geographic area. | User interface classified into two tabs   1. To indicate critically ill patents 2. Notify details of medicine having been administered   Classifying the user interface by using VHF radio connections will be beneficial, especially for early stages of disaster-stricken developing countries |  |
| **Syed-Mohamad et al (2010)**  The development and design of an electronic patient record using open source web-based technology  **(Malaysia)** | Describes the method used to develop an electronic patient record (EPR) for the One Stop Crisis Center (OSCC) and to demonstrate the system’s features and functionalities. A discussion of implementation issues and suggestions for further improvements are also included. | Description of process.  10 participants , two each from five participating departments (Emergency; Obstetrics and Gynecology; Pediatrics; Psychiatry and Social Work) participated in training workshops. | Proved coordination between disciplines and standardization of data in hospital were noticed. It is expected that this will in turn result in improved data confidentiality and data integrity. The collected data will also be useful for quality assessment and research. |  |
| **Tan et al (2009)**  Evaluating user satisfaction with an electronic prescription system in a primary care group  **(Singapore)** | Examine users’ satisfaction and factors associated with an electronic prescription system implemented in the National Healthcare Group Polyclinics in Singapore. | An anonymous survey was administered in October 2007 to all physicians, pharmacists and pharmacy technicians working in the 9 National Healthcare Group Polyclinics. | High degree of agreement that electronic prescribing reduces prescribing errors and interventions, with participant reluctance to go back to the paper-based system.  The results also revealed that satisfaction with the system was more positively associated with users’ perceptions about the electronic prescription system’s impact on productivity rather than on quality of care. | Study captured respondents’ perceptions during a transitional phase and may reflect the impressions of users who are still beginning to learn the system. With time and experience, users’ perceptions of the system may change.  The survey was not designed to assess whether external factors such as implementation, customisation and institutional readiness could influence user satisfaction |
| **Tan et al. (2010)**  Usability of clinician order entry systems in Singapore: an assessment of end-user satisfaction  **(Singapore)** | Determine the overall satisfaction level of NHG users (doctors and nurses) with respect to their order entry systems; and identify the constructs that are associated with user satisfaction. | Fifty doctors and fifty nurses from various institutions under the National Health Care Group (NHG) were randomly selected, with a total of 52 respondents. | Users’ satisfaction with the clinical systems was 3.85 out of 7.  System reliability, intuitive navigational capabilities and ease of use are strongly and positively correlated with user satisfaction. System response time, however, is found to be strongly but negatively correlated with user satisfaction with a correlation coefficient of -0.717 (p<0.001) | The relatively low response rate can lead to selection bias. |
| **Tan et al. (2013)**  An electronic dashboard to improve nursing care  **(Singapore)** | Cost-pilot study utilising data from system usage logs and a survey questionnaire to gather end-user feedback and evaluate the factors that influenced user satisfaction of the Andon system. | A dashboard device which provides simplified visual management of a production line was created. It enabled hospitals to design interactive features and monitor actual usage through the clicks made in real time.  122 nurses from 170 eligible staff responded. 106 included in the study, 16 surveys were incomplete. | Mean satisfaction score was 3.6 out of 5. User satisfaction was strongly and positively correlated to the system’s perceived impact on work efficiency and care quality. From qualitative feedback, nurses generally agreed that the dashboard had improved their awareness of critical patient issues.  An interactive clinical dashboard when properly integrated with a computerised physician order entry system could be a useful tool to improve daily patient care. | Study was designed to gather quick feedback after pilot. Might not be able to fully extrapolate or generalise these results across all institutions. |
| **Thit et al (2016)**  Electronic medical records in Myanmar: user perceptions at Marie Stopes International clinics in Myanmar  **(Myanmar)** | To explore users’ perceptions of EMR in an international non-governmental organisation in Myanmar. | Individual interviews using structured interview questions were held with participants from all levels of the organisation. A proposal of an EHR system was then offered including a definition of EMR, samples and scenarios. | Four key themes highlighted: concerns with current system included time and work duplication; concerns with transition to new system included training and increased workload; perceived challenges with the new system included confidentiality, technical support, unstable power supply and sustainability; and end user perspectives. The study highlighted need for organizational and technical support. |  |
| **Wang et al (2016)**  Exploring physicians extended use of electronic health records (EHRs): a social influence perspective  **(China)** | To use social influence theory to examine the impact of mechanisms of social influence on intentions of physicians to extend their use of EHR. | Survey (n=205) of physicians in a hospital in China 2 years following implementation of an EHR system. A 16-item questionnaire implemented to measure 4 social influences: reward, punishment, social image and group norms. Validity and structural analysis was completed and partial least squares analysis was performed to examine the components. | Significant relationship between physician’s responses on two social influence measures: reward and group norms (internalisation). Punishment and social image did not appear to impact intentions to increase the use of EHR. To maximise implementation hospital management should consider reward and promotion of EHR as a group norm as ways to increase usage of systems. | Data for the study collected at one point in time and may have missed dynamics of extended use. Data collection was in a public hospital in China which may limit generalisability. |
| **Wongsapai et al (2013)**  Health-oriented electronic oral health record for health surveillance  **(Thailand)** | To develop and evaluate a new Health-oriented Electronic Oral Health Record (Health-EOHR) that integrated new oral health status into a graphical user interface, the health-oriented status and intervention model to facilitate oral health surveillance. | Focus groups and a Delphi process to develop health-oriented status and intervention model and graphical user interface. | Overall, the dentists were significantly satisfied with the Health-EOHR compared to the existing EOHR (p < 0.001). The dentists found it easy to use and were generally satisfied with the function and the impact on their work, oral health services and surveillance. |  |
| **Wu et al (2017)**  Implementation of a cloud-based electronic medical records exchange system in compliance with the integrating healthcare enterprise’s cross-enterprise document sharing integration profile  **(Taiwan)** | To build an electronic medical records system (EMR) in compliance with the Integrating the Healthcare Enterprise (IHE)’s Cross-Enterprise Document Sharing (XDS) integration profile.  To propose a feasible cloud-based EMR exchange framework capable of efficient EMR exchange among hospitals and clinics. | Representational state transfer (RESTful) services were used to implement the proposed prototyping system on the Microsoft Azure cloud-computing platform. Four scenarios were created in Microsoft Azure to determine the feasibility and effectiveness of the proposed system. | RESTful service approach is superior to the Simple Object Access Protocol method currently implemented in the National Electronic Medical Record Exchange Center (EEC) system, according to the irrespective response time. | Security mechanisms of EMR during exchange is not proposed in this paper. |
| **Yan et al (2013)**  ISS – An electronic syndromic surveillance system for infectious diseases in rural China  **(China)** | To describe design and pilot implementation of an electronic surveillance system (ISS) for early detection of infectious disease epidemics in rural China. | ISS was developed on an existing platform combined with four interconnected functions: work group and communication group; data source and collection; data visualisation; and outbreak detection and alerting. ISS platform collected daily syndrome information from three different data sources: health facilities, medication sales from retail pharmacies; and primary school absenteeism. | ISS was installed and implemented in four counties in rural China for six months. 95 health facilities, 14 pharmacies and 24 primary schools were included in the study generating 74,256, 79,701 and 2330 daily records into the central database.  The ISS system showed significant advantages over conventional surveillance system with a simple and flexible data reporting system. Some seasonal patterns were identified consistent with seasonal epidemics. | Outbreak modelling system demonstrating feasibility of collecting functional surveillance data. Still needs to be further validated for confirmed outbreaks. ISS collect from pharmacy electronic records, biasing towards bigger pharmacies, which may lead to underreporting—particularly in more rural settings. Quality of surveillance data questioned as EMR systems not widely available, therefore requiring manual data entry. Challenges included a continuation of paper-based record keeping and required manual labour for data entry, leading to labour demand and quality control issues. |
| **Yu et al (2016)**  CSDC - a nationwide screening platform for Stroke control and prevention in China  **(China)** | Outlines the design and implementation of China Stroke Data Center (CSDC) and results of applying this platform. | The platform is composed of data collected from high-risk populations and EMR from individual patients with stroke and a big data analysis system. Patient data included risk factors, diagnosis history, treatment, and sociodemographic characteristics. The platform is divided into 3 layers: data, model and application. Data layer includes data collection, storage and exchange. Model consists of big data analysis and requisite software components. Application includes analysis service interfaces and a web portal. | By 2014, 4.5 million peoples’ screening data and 0.4 million patients’ follow-up data compiled. 47.8% originated from community health (primary care) institutions and 39.4% came from township health institutions (secondary care facilities). |  |

**Appendix Table 2. Summary of thematic analysis of articles included in the review**

| **Authors & Title** | **Roles in public health** | | **Key success factors or potential barriers** | | |
| --- | --- | --- | --- | --- | --- |
|  | **Benefits** | **Level of implementation** | **EMR Context**  **(Overall health system)** | **EMR input** | **EMR Output**  **(Data management, visualization, implications)** |
|  |  |  |  |  |  |
| **Alipour et al (2017)**  Success of failure of hospital information system of public hospitals affiliated with Zahedan University of Medical Sciences: a cross sectional study in Southeast of Iran  **(Iran)** | A hospital information system designed to manage all the aspects of a hospital operations such as clinical, administrative, and financial activities. | Four hospitals associated with Zahedan University of Medical Sciences. | Cultural factors associated with attitudes towards HIS use. Training of staff was also a key factor. | No EMR input in this study, instead assessed end user feedback in public hospitals using key information subsystems in place for 5-10 years prior to this study and include: financial; admission, discharge and transfer; nursing; laboratory; and pharmacy. | Need training courses on factors affecting success or failure of HIS for internal stakeholders, provided before, during and after implementation.  In-service training needed, especially in the case of new software or software upgrades. |
| **Baldwin et al (2008)**  Managing information: using systematic data collection to estimate process and impact indicators related to harm reduction services in Myanmar  **(Myanmar)** | Management Information Systems (MIS) can be broadened to monitor and measure coverage and scale-up of harm reduction services, as well as provide descriptive data on the range and quality of services. | Conducted amongst NGO providers in a low-income country (Myanmar), specifically among several partner agencies implementing harm reduction programs. | Study conducted in the absence of coordinated or integrated monitoring and evaluation systems, and no known existing systems to consolidate data collected at a national level.  Burnet Institute evaluation suggested that quality of M&E systems across harm reduction project sites varied, was not standardized, and that many partners did not use data to evaluate and/or improve services. | Development of MIS took the following into account:   - Clarity of vision; - avoiding complexity; - a “minimalist approach”;   output analysis. | Recommends EHR inputs and visualizations to be based around standardizing unique client identification codes; a modular MIS structure; standardization; flexibility; bilingual interface; and data analysis and reporting. |
| **Chang et al (2015)**  A context aware approach for progression tracking of medical concepts in electronic medical records  **(Taiwan)** | Promotes identification of information relevant to heart disease risks (or potentially other target medical concepts) and track progress to aid in clinical decision making. Develops a system that recognises medical concepts/risk factors including: diabetes, CAD, obesity, hypertension, family history and medication lists. | International, multiple sites in 2 countries. | New algorithms were implemented in places that have worked with EMR systems for some time (Taiwan, Australia). They are supported by faculty and departments with expertise not only in clinical medicine, but also bioinformatics. Data management is in line with systems needed for secure management of EMR. | Tested a dictionary-based and machine-based algorithm for medical concepts as well as numeric, non-numeric and family history risk factors.  Medical/Mention concept analyser was developed to indicate diagnosis of diabetes, CAD, hypertension, hyperlipidemia, and obesity. | Diseases (specifically, cardiovascular disease), associated risk factors, and time attributes related to risk factors and/or disease states or sequelae.  Created online repository of natural language processing components used in this context-aware algorithm. |
| **Eunmi et al (2016)**  The automated alert system for the hospital infection control and the safety of medical staff based on EMR data  **(South Korea)** | Design of a usable, automated infection alert system to document exposure, infection and resolution. Allows sharing of information for patients exposed to infection, reduces re-occurrence and protects staff and patients. | University level (Seoul National University Hospital). | Formerly, EMR used a manual system requiring time and effort of health staff to maintain. | Programme setting for alert system: required infectious disease labels, infectious disease transmission types, automation of lab results, rules of data migration and access to past medical histories.  Infection registration or release can be done manually. | Provides an information page through user interface. The programme provides guidelines for infection control and patient history for user screens (pop up when user keys in “any alert words”). |
| **Herbst et al (2015)**  The INDEPTH data repository: an international resource for longitudinal population and health data from health and demographic surveillance systems.  **(Africa/Asia)** | INDEPTH Data Repository to enable member centers and associated researchers to contribute and share fully documented, high-quality datasets with the scientific community and health policy makers. | International, found health and demographic surveillance systems in 52 low- and middle-income countries, including in Asia (India, Bangladesh, Thailand, Vietnam, and Indonesia). | Contexts highly variable between countries with broad range of funding for services and technological infrastructure. However, focused on low- and middle-income countries. | In addition to staffed support teams, included distribution of “Centre-in-a-Box” (CiB) to each participating center that came equipped with portable mini-server hardware, database servers; and data manager workstations.  Developed standardized analytical datasets across sites through common data specification.  Workshops training data managers in the use of CiB.  Quality assurance by applying minimum data quality metric; summary indicators; plausibility review.  A final approval stage allows for data producer agreements with the INDEPTH Data Access and Sharing Policy and agree to a specified data access level. | Pentaho Data Integration software programme used to extract data from the different underlying database systems, transform the data into a standard format, and load data into the repository.  Nesstar Publisher used to prepare data documents compliant with standards.  System servers: Zentyal, a Linux-based server manging network security, user authentication, and a shared file system for the CiB.  Microdata Cataloguing Tool to view data documentation prior to uploading the documentation to the network repository.  Repository also based on web-based content management application. |
| **Kimura et al (2011)**  Developing an electronic health record for intractable diseases in Japan  **(Japan)** | Intractable diseases are difficult to distinguish from diseases with a similar clinical profile; these diseases may also alter in clinical patterns over the long term, so there is a need to have life-long health records and standardised clinical information models to allow for better research and characterization of these diseases. | National (Ministry of Health, Labor, and Welfare).  Collected and collated clinical information throughout the country specific for intractable disease. Automated and digitised records used when available. | Existing system allows for passive detection of intractable diseases through health care subsidies patients receive through their prefecture. At the time of the present study, some hospital systems with existing EMR. | Computerised clinical research form; EHR set up at data center at National Institute of Public Health. Allowed for submission from healthcare workers. Clinical research entry form given to participating hospitals. Developed unique document identifiers for each form. Developed QR coding system to allow anonymising sensitive patient information.  An automated registration form was also designed. | New system enabled searching EHR according to archetype. Data mapping possible but required trial-and-error approach.  Sets up unique identifier system. |
| **Lee et al (2015)**  Validation for accuracy of cancer diagnosis in electronic medical records using a text mining method  **(South Korea)** | Validation of accuracy of diagnosis allows tracking of disease. Can be utilised for diseases requiring pathological diagnosis such as cancer. | Asan Medical Center, South Korea (Provincial level). | Research conducted in system with existing and implemented EMR. | Existing EMR with pathology reports and data entry (ICD 10) completed by the clinician.  Used a text mining approach/algorithm for detection of key pathology terms to compare against clinical diagnosis. | Suggests a systematic alarm and feedback to clinicians to support correct data entering and diagnosis. |
| **Li et al (2013)**  e-Health preparedness assessment in the context of an influenza pandemic: a qualitative study in China  **(China)** | Pandemics place significant burden on health services. e-Health services may allow more effective functioning of healthcare facilities.  Assessment of organisational systems and preparedness critical for future e-Health interventions. Need to identify resistance to change at individual and organisational levels. | Provincial/Municipal level (Beijing). | Study conducted in place with no EMR system. However, IT team in place for HIS management and ICT systems utilising clinical and non-clinical software. IT systems dating back to 1999, with key updates in 2003. | Manual input of data from paper-based surveillance forms submitted to the CDC (from district to province and national level). | Key needs in preparing e-Health interventions: informative and efficient sharing of patient records; potential automated checks for prescription errors; use of reference portals; awareness raising of clinicians to engage and use EHR systems, appropriate user-friendly interfaces.  Between each level, the data are shared via website (intranet of CDC). |
| **Liabsuetrakul et al (2016)**  Development of web-based epidemiological surveillance system with health system response for improving maternal and newborn health: field-testing in Thailand  **(Thailand)** | To test the acceptance and intention to use health information technology that is being developed and/or designed. | Provincial level, Songkhla province Thailand.  Specifically in labor and delivery wards of 8 hospitals in a southern province of Thailand. | Implemented in Thai system with strong mobile technology infrastructure (3G compatible).  Interoperability through different levels within the provincial public health system. | Web-based application designed to meet ease of access and flexibility in operating systems.  Three levels of database access executed: data entry operator, actor, and administrator.  Functional design with training for operators as well as systems that utilized smartphones, pocket PCs, or desktop computer.  Three levels of data security: client, data transmission, server.  Automated analysis; automated notifications for unconfirmed data. Manually added complications within 24 h of delivery. | Allowed for access to all individual data, action responses and aggregated summaries of the data from all hospitals including result presentations in tables, graphs, and maps (Administrator level database).  Visualized five options on main screen, with 10 complications listed; complications requiring action were highlighted. |
| **Lo et al (2014)**  Improving the work efficiency of healthcare-associated infection surveillance systems using electronic medical records  **(Taiwan)** | Utilising EMR systems for improving infection control. | University hospital in Taiwan. | Compared to an older system (an electronic culture-based surveillance system), new system found improvement in time associated with infection control professionals work and increased sensitivity in case finding. | Auto-detect from six variables available within hospital culture (EMR).  Inputs include an integrated, automated system that allowed for case detection through 6 variables that provide discriminant score; extracted surveillance data from EMR systems; with case management functions to monitor and update patients’ infection status.  Features automated reporting to Taiwan CDC. | The visualization dashboard has integrated tubular view (allowing access to complete information of the patient).  Automated system detects infected patients and feeds to infection control professionals for decision-making.  Infection Control Professionals with additional access through laptops and mobile phones.  National surveillance possible through automated reports to central, Taiwan CDC. |
| **Low et al (2017)**  Evaluation of a practical expert defined approach to patient population segmentation: a case study in Singapore  **(Singapore)** | Segmenting the population into groups that are relatively homogenous based on healthcare needs can facilitate the planning for resource allocation and the design of integrated care programmes or practice units around patient segments. | National.  Used existing SingHealth RHS data for a complete year. | EHR used for SingHealth, well established in Singapore. Integrates information from multiple sources including administrative data, clinical data and ancillary data. | Represents a retrospective review of case records within an EMR/EHR system. | Can allow for reporting on trends and prediction models through use of EHR of RHS in future works.  Implications for disease control, health behavior, and biopsychosocial needs and coordination of care.  Allowed for a continuous iterative process to refine segmentation based on patient-centered needs and inform the design of tailored bundles of care. |
| **Low et al (2017)**  FAM-FACE-SG: a score for risk stratification of frequent hospital admitters  **(Singapore)** | Developing scores based on EHR that identify potential high-cost users or frequent hospital admitters. | National, tertiary level hospital.  Retrospective review of EHR data at Singapore General Hospital, through SingHealth. | EHR system nationally through the SingHealth RHS. | No specific EMR/EHR input performed as part of this study.  Developed a model for risk stratification using 70% of EHR patient data for derivation and 30% of EHR patient data for validation. | Significantly better performance of the Singapore-specific model for risk stratification developed here compared to the LACE index.  FAM-FACE-SG has near real-time availability early in admission.  Implications to drive implementation/adoption is automation: all variables can be retrieved from the EHR near real-time and can be automated into a risk score to be fed back to clinicians and case managers. |
| **Low et al (2017)**  Performance of the LACE index to identify elderly patients at high risk for hospital readmission in Singapore  **(Singapore)** | To identify high-risk, elderly populations and risk of readmission, which can strain limited healthcare resources and lead to increased healthcare costs through unplanned readmissions. | National level, tertiary care hospital (Singapore General Hospital).  Utilized EHR data collected through SingHealth. | EHR system nationally through Singapore General Hospital and SingHealth system.  System developed in 2012. | No specific EMR/EHR input.  Retrospective analysis of cohort of data collected from existing EHR system.  Set up risk models through LACE framework and compared models augmented with additional risk factors. | Implication for existing models such as LACE with poor discriminative ability within the Singapore context.  Implies further research needed for developing better performing models based on Singapore data routinely collected, such as clinical, social, and functional variables with costs and labor factors taken into consideration. |
| **Mohd Salleh et al (2016)**  The influence of system quality characteristics on health care providers’ performance: empirical evidence from Malaysia  **(Malaysia)** | Evaluates HIS in hospitals in how they contribute to the performance of health care providers given the considerable investment by governments to improve and mandate these systems. | National.  Used largest public hospital in Malaysia. | EHR systems in place in Malaysia at the time of this study. | Performed an evaluation of provider performance based on adapting the EHR System Effectiveness Models. | Contribution to IS theories.  Provide practical measures for system quality and positive impacts on individual health care providers’ performances.  Implies that hospital management might enhance their annual IT allocation budget and strategically plan for future system’s development by considering technology characteristics related to clinical task-technology compatibility. |
| **Murai et al (2011)**  Systemic factors of errors in the case identification process of the national routine health information system: a case study of Modified Field Health Services Information System in the Philippines  **(Philippines)** | Evaluation of health worker understanding in compiling high quality data within HIS as a significant component in overall data quality systems and evidence-based practice based on collected data. | Provincial; Palawan province in the Philippines. | Used the Field Health Services Information System developed in 1996 in the Philippines (existing EHR). | No proposed EMR/EHR interventions.  Questionnaire provided to health workers to assess their understanding of coding definitions to be interpreted as possible gap in data quality due to health worker error. | Highlights causes of confusion around potential coding errors of the health workers:  Clarifying indicators that are unsupported by the current conditions in the health system.  Clarifying indicators with incomplete or ambiguous definitions.  Identifies indicators with complete definitions but easily misunderstood by health workers.  Attention to these systemic factors that may relate to health worker errors can improve data quality. |
| **Nguyen** **et al (2013)**  A method to manage and share anti-retroviral (ARV) therapy information of human immunodeficiency virus (HIV) patients in Vietnam  **(Vietnam)** | Manage and share anti-retroviral (ARV) therapy information of human immunodeficiency virus (HIV) patients in Vietnam. | Three health care centers in Hanoi. | Lack of infrastructure (computers, connectivity).  Lack of trained workforce with computer proficiency. | Standardization of template and data format into 32 elements (covering patient information, ARV treatment and blood examination results).  Backup tool provided and further backed up on the external device on monthly basis.  Three tier security system: 1) individual username and password, 2) system controls to authorized access to user groups and user purposes 3) administrator to control and monitor use access.  Data encryption and decryption.  Centralized database connected through intranet.  Decentralized management, remote clinics table to track and manage patients data as well as update information. | Use MVC framework (Model View Controller) with three components.  Model: manages data, responds to information requests.  View: generates outputs to users and display with the use of Graphic User Interface (GUI).  Controller: interpret/translate user inputs and instructs further operations accordingly. |
| **Rachmani et al (2013)**  Health information system model for monitoring treatment and surveillance for Leprosy patients in Indonesia (case study in Pekalongan District, Central Java, Indonesia)  **(Indonesia)** | Monitoring for patients with leprosy infections, which requires an extended therapeutic regimen. | District-level facilities. | Issue of rotating staff causes discontinuity with patient records as well as routine follow up.  Article implies HR constraints in current and future computerised systems. | Manual data entry using Microsoft Excel (limits the treatment monitoring).  Implementation of computerised databases that can follow leprosy patients and monitoring trends. | Computerised databases on leprosy including data collection, reports and analysis as well as predictions and estimates are needed. |
| **Radhakrishna et al (2014)**  Electronic health records and information portability: a pilot study in a rural primary healthcare center in India  **(India)** | EHR help gather community-level database (sociodemographic and medical profile). Can be transferred between community health centres and hospitals by USB device. | District level (villages in rural India). | Routine medical records are still paper-based.  Issues of access to healthcare between urban and rural areas.  Low literacy rates but good access to mobile phone technology.  Fragmented healthcare system with public and private providers.  Lack of funds for healthcare IT infrastructure, technical standards and initiatives from public health governance. | The data were transcribed manually to EHR. Then connected to USB and SMS system.  USB cards: Personal health data could only be entered/updated at point of entry.  SMS messages with four categories of accessible data: demographic, problem list, last investigation results and latest prescription list. | Data visualization read-only outside home clinic where patient initially registered.  Password-protected USB card linked via request code(s) to the valid phone number registered to EHR to help secure patient confidentiality.  Mobile- based SMS systems allow patients access to their own health information as needed. This also helps evaluate health data-seeking behavior. |
| **Jinpon et al (2017)**  Integrated information visualization to support decision-making in order to strengthen communities: design and usability evaluation  **(Thailand)** | CWBAS potential tool for measuring community well-being, tracks changes over time and identifies policy measures to improve community outcomes. | National. Sub-districts representing all 4 regions in Thailand.  CWBAS implemented at the sub-district and village levels. | Leveraged existing framework and web-based technology of the CWBAS, using data obtained as part of the Family and Community Assessment Program (FAP) which was developed using Microsoft Access.  Existing CWBAS developed using web-based technology, Business Intelligence concepts, open sourcing computing environments (MySQL, PHP, Highcharts JS, Google Maps). | Implemented data visualization and GIS tools.  Data entry system designed to check for consistency and accuracy and allowed users to preview data prior to adding to the system. | Dashboard that allowed for graphical analytic reports; visual and real-time representation of aggregated data around community well-being; and advanced statistical tools. |
| **Rahman et al (2015)**  Emergency medical services key performance  measurement in Asian cities  **(Asia)** | Compares EMS performance index by structure, process and outcome analysis to outline ways forward in EMS performance indices for comparison and benchmarking. | Asian cities (Tokyo, Osaka, Singapore, Bangkok, Kuala Lumpur, Taipei and Seoul). Presents some national level data from participating countries. | Variability in maturity of EMS setups across Asian cities in the region. Although many countries have robust data systems, lack of uniform and standardized data collection.  Abstracting information from paper forms is difficult, which remains common pre-hospital care provision. | No specific intervention implemented.  Feedback from surveys given to representatives of participating EMS agencies or medical directors of the Pan-Asian Resuscitation Outcome Study (PAROS) group. | Variability in core measure definition.  Need for consensual common taxonomy and data collection methodology to create uniform EMS measures in the region and identify common available core measures from each country/cities. |
| **Samy et al (2010)**  Security threats categories in healthcare information systems  **(Malaysia)** | Progression to paperless and film-less operation in all aspects of the hospital’s operation. | A single government hospital.  Structured interviews with key health staff, including IT staff. | Participants taken from tertiary care hospital in Malaysia equipped with Total Hospital Information System (THIS). | Human error (erroneous data input, deletion or modification of data). | Highlights 5 potential threats to THIS: power failure/loss, human error;  technological obsolescence,  hardware and software failure. |
| **Sutiono et al (2010)**  Designing an emergency medical information system for the early stages of disasters in developing countries: the human interface advantage, simplicity and efficiency  **(Indonesia)** | Assessing minimal contents of user-based interfaces for ease of use in emergency medical care in early stages of disasters. | Garut City, Indonesia. | Usage of VHF (very high frequency) radio modems allocated for use in disasters. Emergency medical care information system (EMCIS) supported in study pilot area. Included infrastructure for wireless transmission of data through EMCIS. | Development and implementation of EMCIS.  Design user interface analysis and considered common scenarios.  Prototype systems architecture including ICT hardware and software as well as electronic databases. | EMCIS that includes patient demographics; triage for severity; support and treatment management; head-to-toe examination. |
| **Syed-Mohamad et al (2010)**  The development and design of an electronic patient record using open source web-based technology  **(Malaysia)** | Coordination between disciplines and standardisation of data in hospital is expected to result in improved data confidentiality and data integrity. The collected data will also be useful for quality assessment and research. | Hospital Universiti Sains Malaysia (HUSM) in Kelantan, Malaysia. | Patients informed that their data are stored in the system, patient consent at an early stage in the process of patient care required.  Utilisation of web-based open source software (any browser/any computer).  No uniform format for paper-based forms across departments. | Mixed language (English and Malay) to improve and facilitate the existing work flow.  Users can only access modules that have been authorised to them.  User login and interventions to systems are recorded by time stamping.  System is capable of producing log sheets.  Assessed attitudes toward computers. | Individual patient reports to any caregivers.  Periodic statistical analyses can only be accessed by OSCC coordinator. |
| **Tan et al (2009)**  Evaluating user satisfaction with an electronic prescription system in a primary care group  **(Singapore)** | Evaluating an electronic prescribing system through user feedback.  Assesses users’ satisfaction with system functionality, system’s impact on productivity and the perceived impact on prescription errors and interventions. | 9 National Healthcare Group polyclinics.  Survey of physicians and pharmacy staff of electronic prescribing system implemented in 2006. | Singapore government has made health information technology (IT) a cornerstone to delivery of personalised health care services for high quality and cost-effective clinical care. Sufficient funds to develop the IT infrastructure necessary for EHR systems. | Computerised Physician Order Entry (CPOE) introduced in 2006.  System maintains:   - Complete medication list; - Clinical decision-support tools (alerts/reminders); - Two-way electronic communication between doctors and the pharmacy. | Respondents with positive feedback on functional features of the system: alerts for drug-drug interactions or drug allergies; creation of new or amend processed prescriptions; time taken to enter prescription information; detection of errors.  Computer literacy perceived to be high among participants, correlating with ability to use electronic prescription system. However, prior computing knowledge not associated with overall satisfaction.  Pharmacy staff satisfied with downloading of new prescriptions via pharmacy computers.  Participants perceive sufficient training conducted before launch of the system. |
| **Tan et al (2010)**  Usability of clinician order entry systems in Singapore: an assessment of end-user satisfaction  **(Singapore)** | To gather and evaluate end-user feedback on factors related to satisfaction with Computerised Physician Order Entry (CPOE).  Improvement of CPOE to influence clinician satisfaction and adoption. | National Healthcare Group (Alexandra Hospital, NHG polyclinics, The National University Hospital and Tan Tock Seng Hospital). | The National Health Group in Singapore designed and implemented the CPOE system for laboratory, radiology and medication orders in the four years prior to this study. | Review of existing CPOE system. | Features of the CPOE reviewed favorably: remote access to clinical information; information compiled in one accessible “place;” ability to monitor laboratory results.  Automated alerts for medication administration.  Dissatisfied with system response time  Overall, user satisfaction relates to four key factors: system reliability; intuitive navigational capabilities; ease of use; system response times.  Highlights need for optimizing clinical software design and improving and maintaining IT infrastructure. |
| **Tan et al (2013)**  An electronic dashboard to improve nursing care  **(Singapore)** | Evaluation of nurses using specialized nursing dashboards on top of existing CPOE system.  Potential for improving communication across multidisciplinary teams, coordination of patient care; prevention of clinical/treatment errors. | Khoo Teck Puat Hospital, Singapore. | CPOE system introduced in 2009. Dashboard developed in addition to the existing CPOE system. | Evaluating user feedback of nursing dashboard developed and implemented in 2011. Assessed: overall user satisfaction; usage frequency; system quality; system information quality; impact on work efficiency; impact on care quality.  Dashboard allowed for simplified visual management of a production line. An interactive dashboard using touch screen computers was then piloted. | Dashboard aimed to flag and alert key patient issues such as urgent orders; abnormal lab or radiology results; infection control alerts.  Various icons on screen to bring up pertinent information.  Allowed quick review of all critical patient tasks and alerts via one screen (bypassing hospital EMR).  High utilisation of features that improved communication between nurses and the radiology department. |
| **Thit et al (2016)**  Electronic medical records in Myanmar: user perceptions at Marie Stopes International clinics in Myanmar  **(Myanmar)** | EMR systems can help identify records and maintain important patient information such as medical history, demographics, problem and medication lists, clinical documents and notes, care plans and generate and record patient-specific instructions. | Organisational level (international non-governmental organisation). | Myanmar currently without wide-scale, EMR system.  Private funding makes it possible to have EMR implementation in some organisations. | No intervention/input for EMR/EHG used in this study; study of perceptions of such systems among potential users.  Patient’s files will contain patient’s information and care plan which can be retrieved and removed any time. | This study suggests feasibility assessments should be undertaken to assess technical support, training, and infrastructure needed for a sustainable EMR system. Highlights the need to address staff confidence in using ICT.  Future EMR outputs should consider: HR and employee capacity development, ICT literacy and IT technical support; ensuring patient confidentiality and regular access to the EMR system. |
| **Wang et al (2016)**  Exploring physicians extended use of electronic health records (EHRs): a social influence perspective  **(China)** | EHR, the more advanced stage of EMR, are for medical data sharing among stakeholders in accessing health information.  Understanding behaviours and incentives for use of EHRs may be helpful in roll-out and implementation of EMR/EHR elsewhere. | Regional level (Southern China). | Study takes place as an evaluation within a large, tertiary care facility after 2 years of EHR implementation. Existing EHR comprised of four modules: health information data; order entry and support; results management; and decision support. | No intervention or input for EMR/EHR used in this study. | Study confirmed that physicians’ intentions to use EHR was influenced by socially influenced factors. These included rewards and group norms. Practical implications for EHR implementation and management of healthcare organisations in extending use of EHR. |
| **Wongsapai et al (2013)**  Health-oriented electronic oral health record for health surveillance  **(Thailand)** | Identification of the size and character of oral diseases and the need for oral healthcare at individual and community levels. The potential to empower decision-makers to lead and manage more effectively by providing useful oral health records | 26 dentist from 11 government hospitals. | Currently designing EMR input as an open-source Health-EOHR that can plug into existing Hospital Information System. | Development of a Health-oriented Electronic Oral Health Record (Health-EOHR) integrating health-oriented status and intervention index to facilitate planning, managing, and evaluating healthcare delivery system.  Assessment of end-user feedback. | Each oral health status linked with suggested intervention, instrument, costs, intervention time and personnel needed. |
| **Wu et al (2017)**  Implementation of a cloud-based electronic medical records exchange system in compliance with the integrating healthcare enterprise’s cross-enterprise document sharing integration profile  **(Taiwan)** | Improving EMR utilising a Cross-Enterprise Document Sharing (XDS) integration profile to an existing, national EMR system for more efficient and safe EMR exchange among hospitals and clinics. | National | Significant number of domestic hospitals within Taiwan already with EMR systems in place. Ministry of Health and Welfare created and EMR Exchange Center (EEC) in 2011 to allow for efficient exchange and ensure integrity and accuracy of data. | EMR exchange system implemented on Microsoft Azure cloud-computing platform to be transferred on other cloud computing platforms.  Security mechanism of electronic medical records during exchange  Performed multiple scenarios for various, designed actors within an experimental environment and evaluated the response time performance for each proposed systems. | Utilised visualization technologies and dynamic resources on demand implemented through the Microsoft Azure cloud-computing platform. The architecture proposed is compliant with the XDS integration profile and is more flexible than the existing EEC system, has better response time through RESTful services. |
| **Yan et al (2013)**  ISS – An electronic syndromic surveillance system for infectious diseases in rural China  **(China)** | EHR systems allow timely identification of outbreaks and disease patterns based on syndromic surveillance. | Provincial level. Rural areas in China. | EMR are not available in most health departments in rural China requiring manual labor to transfer data from paper-based form to electronic databases. | Supervision and monitoring of data collection from paper records and transfer to electronic database.  The data were automatically checked by the system once transferred to the central database which need resubmission after correction.  Users can input, review, query, export, analyse and visualise surveillance data through the password-protected, encrypted website (with different authorities). | Developed interface platform with flexible system and customizable settings.  Different types of data streams require a complex multi-sector strategy and cooperation between data stream owners. Equally poor infrastructure for electricity supply in rural areas constrains the development of a web-based system. Power outages and internet challenges implicated in data reporting issues. |
| **Yu et al (2016)**  CSDC- a nationwide screening platform for Stroke control and prevention in China  **(China)** | Data integration allows the collection of data on risk factors, diagnosis history, treatment and socio-demographic characteristics. This should allow the provision of information for clinical decision making and evaluation, research and public health services.  EMR is used as one part of the nationwide data integration and analysis system (focusing on stroke). | National. | National web-based system built to maintain screening data from high-risk populations and EMR from individual patients with stroke to inform China stroke screening and intervention programme. | Created platform to compile patient data for stroke cases with risks.  Data collection by submitting integration form online. Screening data from high risk populations and individuals with stroke then validated by web-based system, hospital data administrator and randomly checked (5%) for quality control. Compiled follow-up for in- and outpatient data. EMR data from hospitals analysed through China Stroke Data Integrator (CSDI) under encryption technology. | CSDC system designed in 3 layers. Includes data (integrated and exchange system), model (big data analysis) and application (analysis interfaces provided by model layer).  CSDI (link hospitals’ EMR to nation stroke database in CSDC) - supports many database systems to make it compatible with heterogeneous data sources.  Online data analysis is available. |
